# Measuring the health of the Indian elderly: evidence from National Sample Survey data

**DOI:** 10.1186/1478-7954-8-30

**Published:** 2010-11-16

**Authors:** Bradley Chen, Ajay Mahal

**Affiliations:** 1 Department of Global Health and Population, Harvard School of Public Health, Boston, USA; 2 Department of Epidemiology & Preventive Medicine, Monash University, Melbourne, Australia

## Abstract

**Background:**

Comparable health measures across different sets of populations are essential for describing the distribution of health outcomes and assessing the impact of interventions on these outcomes. Self-reported health (SRH) is a commonly used indicator of health in household surveys and has been shown to be predictive of future mortality. However, the susceptibility of SRH to influence by individuals' expectations complicates its interpretation and undermines its usefulness.

**Methods:**

This paper applies the empirical methodology of Lindeboom and van Doorslaer (2004) to investigate elderly health in India using data from the 52^nd ^round of the National Sample Survey conducted in 1995-96 that includes both an SRH variable as well as a range of objective indicators of disability and ill health. The empirical testing was conducted on stratified homogeneous groups, based on four factors: gender, education, rural-urban residence, and region.

**Results:**

We find that region generally has a significant impact on how women perceive their health. Reporting heterogeneity can arise not only from cut-point shifts, but also from differences in health effects by objective health measures. In contrast, we find little evidence of reporting heterogeneity due to differences in gender or educational status within regions. Rural-urban residence does matter in some cases. The findings are robust with different specifications of objective health indicators.

**Conclusions:**

Our exercise supports the thesis that the region of residence is associated with different cut-points and reporting behavior on health surveys. We believe this is the first paper that applies the Lindeboom-van Doorslaer methodology to data on the elderly in a developing country, showing the feasibility of applying this methodology to data from many existing cross-sectional health surveys.

## Background

Improving and maintaining population health are considered important and agreed-upon objectives of health systems [1,2]. Not only is the average level of health important, the issue of health inequalities has also been prominent on the policy agendas of national governments and international organizations [3-6]. In particular, with a growing share of the elderly in developing countries such as China and India, the health of older populations constitutes an issue of growing policy importance.

A reliable and comparable measure of health is necessary for undertaking health inequality studies or any investigation of the impact of policy interventions on population health. Mortality indicators, such as life expectancy and infant mortality rate, do not adequately capture the morbidity aspect of health. Thus, many surveys rely on aggregate measures of self-reported health (SRH), usually as categorical responses, which are easy to administer and have been shown to have good predictive power for subsequent mortality [7,8]. However, the interpersonal, intertemporal, and interregional comparability of SRH is questionable if people understand and respond to questions on the state of their overall health in different ways. Specifically, consider a mapping from an underlying latent health variable (that reflects true health) to an appropriate SRH response category, where the cut-point thresholds at which individuals transit from one categorical response to the next systemically vary across populations [9,10]. Without correcting for those cut-point differences, comparisons based on SRH will lead to misleading conclusions.

This paper assesses the health of the elderly in India using data from the National Sample Survey (NSS) collected in 1995-96, a nationally representative survey of nearly 120,000 households, including about 34,000 elderly persons aged 60 years and above. Our analysis investigates the role of reporting heterogeneity in self-assessed measures of health and tests whether reporting patterns vary by individual characteristics, using responses to additional questions on disability, hospitalization, and chronic disease status in the survey. For this purpose, we use a method developed by Lindeboom and van Doorslaer, who devised tests for assessing whether variations in SRH responses are due to differences in objective health or reflect reporting heterogeneity due to individual attributes such as age, gender, and socioeconomic status [11].

Our paper adds to the literature that aims to better interpret SRH indicators commonly collected in health-related surveys and make the best use of existing information instead of proposing methods to be employed in future surveys, often at enormous expense. For instance, in their classic work, King and colleagues proposed an "anchoring vignettes methodology" to assess and correct for the cut-point shift in individual responses [12], an approach adopted by the World Health Organization in pilot studies of the World Health Survey [9]. Though intuitively appealing and theoretically promising, the anchoring vignettes approach is likely to be difficult to administer in large-scale surveys. Nor is this approach helpful to researchers analyzing and interpreting results already collected from existing surveys, often conducted at great cost in resource-constrained settings. For the vignette approach to yield an unbiased assessment, one still has to assume that respondents use the same sets of cut-points in evaluating the fictitious vignettes and their own health.

This paper also contributes to research on the health of the elderly in India by analyzing a large, nationally representative household survey. The few existing studies on the health of the Indian elderly that we were able to identify focused on small samples and local populations [13-15]. There is one exception, which also used the National Sample Survey data of 1995-96 to assess the relationship between a range of individual attributes and an indicator variable showing whether an individual had a disability or a chronic condition, using a probit regression model [16]. Our approach is differentiated by our focus on assessing the relationship between SRH and more objective indicators of health status available in the survey, and the role that socioeconomic characteristics play in modifying this relationship. Our study also contributes to understanding interregional differences in how individuals report their own health status, such as the paradox highlighted by Amartya Sen, who noted that the people of Kerala reported worse health than the people of Bihar, India's poorest region, even though the former enjoyed much higher levels of life expectancy [17].

## Methods

Following Lindeboom and van Doorslaer [11], we modeled the link between true health status H* and a vector of objective measures of health *H*^*o *^as follows:

(1) $$H_{i}{}^{*}=f(H_{i}{}^{O},Z_{i},\varepsilon_{i})=g(H_{i}{}^{0},\alpha )+\theta Z_{i}+\varepsilon_{i}$$

For any individual "i", let Z denote a collection of explanatory variables that influence an individual's true health state in addition to any objective indicators of health. Note that *θ *and *α *are (vectors of) parameters to be estimated, and ε is an error term. The true health status $H_{i}{}^{*}$ is not observable. Instead, categorical responses *(H*^*s*^) of the following form are observed:

(2) $$\begin{array}{l} H^{S}=k,\;\;\text{when}\;\tau_{k-1}\leq H^{*}<\tau_{k}\;k=1...\text{n},\\ \text{with}\;\tau_{0}=-\infty ;\tau_{n}=+\infty ,\;\;\text{and}\;\;\tau_{ki}={\beta^{'}}_{k}X_{i} \end{array}$$

Here, *τ*_*k *_are the cut-points, with k = 1...n, with cut-points depending on individual characteristics X and parameter vector β. Combining (1) and (2), we get

(3) $$\begin{array}{l} H^{S}=k,\;\;\text{when}\\ {\beta^{'}}_{k-1}X_{i}-\theta Z_{i}-g(H_{i}{}^{O},\alpha )\leq \varepsilon_{i}<{\beta^{'}}_{k}X_{i}-\theta Z_{i}-g(H_{i}{}^{O},\alpha ),\\ \tau_{0}=-\infty ;\tau_{n}=+\infty \end{array}$$

We assume that *ε*_*i *_is distributed as $N(H_{i}^{*},1)$. Equation (3) then describes a standard hierarchical ordered probit model, characterized by cut-points *τ *that depend on individual characteristics, but not on an individual's objective health measures. The likelihood function is given by *ℓ*(*ε/β,θ,α*), the product of the probabilities of observing categorical responses for individuals.

Without exclusion restrictions that force Z to be different from X and without a specific functional form for *g*(.), there is no way to identify the β parameters *separately *from the θ parameters. When X and Z are the same, all we can measure are *β*_01_-*θ*_0_, *β*_02 _- *θ*_0_,... *β*_0*n*-1_-*θ*_0 _for the intercept terms, *β*_11 _- *θ*_1_,... *β*_1*n*-1_-*θ*_1 _for the coefficients of the first term of the vector *X, β*_21_-*θ*_2_,... *β*_2*n*-1_-*θ*_2 _for the coefficients of the second term of the vector *X*, and so on. Thus, as specified, it is not possible to separate shifts in cut-points and the effect of X variables on the true health status H*.

Lindeboom and van Doorslaer [11] described a test for cut-point shifts, by testing the model in (1) and (3) for the restriction *β*_11 _= *β*_12 _= *β*_13 _= ... = *β*_1*n*-1 _= *β*_21 _= ... = 0. If the null hypothesis of restrictions was to be rejected, differences in reported health could be explained as arising at least partly from nonparallel shifts in cut-points across individuals as a result of different X. However, imposing these restrictions in the above specification does not affect parameter estimates if Z and X are the same, and there is no difference in estimates based on the restricted and the unrestricted likelihood functions. Moreover, there is the problem of what happens if we cannot reject the null, given our interest in separating the relative impact of cut-points versus true health effects stemming from the X variables. This means obtaining the estimates of β parameters (cut-point effect) and θ (true health effect) separately, instead of the feasible β-θ.

To address these concerns, Lindeboom and van Doorslaer suggested an approach that relies on stratifying the data so that all intervening variables that influence the relationship between the true health status H* and objective measures of health *H*^*o *^are eliminated from the specification. This means drilling down to fine subgroups where objective measures of health can be treated as being the sole determinants of the true indicator of health, and the cut-points are the same for all members within a subgroup. Thus, if gender, place of residence (rural versus urban), and age-group influence H* separately from *H*^*o*^, we can limit our attention to subgroups such as women over age 60 living in urban areas in the Northern region.

Consider one such subgroup 'm', such that $H_{m}^{*}=g(H^{O},\alpha_{m})$. Within this subgroup, any differences in categorical responses related to SRH can be described by

(4) $$\begin{array}{l} H_{m}{}^{S}=k,\;\;\text{when}\;\;\tau_{m,k-1}\leq H_{m}{}^{*}<\tau_{m,k},\\ \tau_{m0}=-\infty ;\tau_{mn}=+\infty \end{array}$$

Alternately,

$$\begin{array}{l} H_{m}{}^{S}=k,\;\;\text{when}\;\;\tau_{m,k-1}\leq g(H^{O},\alpha_{m})<\tau_{m,k},\\ \tau_{m0}=-\infty ;\tau_{mn}=+\infty \end{array}$$

Undertaking the estimation exercise - maximizing a likelihood function resulting from (3) - for each group, we end up with separate cut-points *τ*_*m*_, and separate estimates of the parameters of the *g*(.) function that generates the categorical responses, namely *α*_*m*_, except for any intercept terms that cannot be separated from parallel shifts in the cut-points. A normalization that constrains the value of any intercept term in *g*(.) to be zero could address this issue, given that it will not affect the actual categorical responses of the household.

Lindeboom and van Doorslaer suggested the following tests for differences in cut-points and *α*. Specifically, consider two groups *m *and $\hat{m}$. In the first instance, one can estimate *τ *and *α *separately for the two groups and calculate their likelihood function values using a standard ordered probit model, given that cut-points *τ *_*m *_and *α *_*m *_are identical for all individuals within each group. Let *L*^*U *^be the sum of the log likelihood values of the unrestricted models. Next, one can construct a joint likelihood function by combining data for the 2 subgroups, using the restriction that $\tau_{m}=\tau_{\hat{m}}$ and $\alpha_{m}=\alpha_{\hat{m}}$. Let *L*^*R *^be the log likelihood value of the restricted model. Then the LR test statistic -2(*L*^*R *^- *L*^*U*^), which is asymptotically distributed as *χ*^2 ^with degrees of freedom equal to the number of restrictions (or the difference in the number of parameters estimated under the restricted and the unrestricted maximum likelihood approaches) can be used to test for restrictions.

When working with data pooled from both subgroups, we could work with either one of two specifications in (5).

(5a) $$g(H^{O},\alpha )=\alpha_{1}H^{O}+\alpha_{2}{\left(H^{O}\right)}^{2}$$

(5b) $$g(H^{O},\alpha )=\alpha_{1}H^{O}+\alpha_{2}{\left(H^{O}\right)}^{2}+\theta D$$

D is a dummy variable indicating membership of the two subgroups. Note that (5b) is more general than (5a) in that it allows for an independent effect of social group membership on true health status. Therefore, we used specification (5b) in our analysis. Effectively, estimating the restricted model means combining the datasets for the two groups *m *and $\hat{m}$, and then carrying out the necessary maximum likelihood estimation exercise.

If the null hypothesis is rejected via the above-mentioned Likelihood Ratio test when specification 5(b) is used, there could be one (or both) of two possible causes - differences in *τ *(nonparallel changes in the cut-points) and/or differences in *α *(differences in the function *g*(.) across the two subgroups). Note that we cannot test for a parallel cut-point shift, as this is not separately identifiable from a shift in the true health effect of the specific subgroup, as captured by the parameter *θ*. To assess whether it is differences in *τ *or differences in *α *(or both) that drive the observed results, Lindeboom and van Doorslaer suggested additional LR tests. To fix ideas, let the log likelihood function for the case where the restriction is solely $\tau_{m}=\tau_{\hat{m}}$ be denoted by *L*^*Rτ *^and for the case where $\alpha_{m}=\alpha_{\hat{m}}$, let the associated value of the log likelihood function be *L*^*Rα*^. Now we can test whether there are nonparallel shifts of the cut-points using the statistic -2(*L*^*R *^*- L*^*Rα*^), with degrees of freedom equal to the number of cut-points times the number of subgroups less the number of cut-points, as the latter would be estimated under the case where all parameters are restricted to be equal across groups. Essentially, we are comparing the null-hypothesis where the cut-points are the same (holding constant the other parameters), versus an alternative where they are not.

To test whether $\alpha_{m}=\alpha_{\hat{m}}$ is valid, we could use the test statistic -2(*L*^*Rα *^- *L*^*U *^), which assesses the difference between no restrictions at all and the case where the *g*(.) function is the same. In specification (5b), to allow for an independent health effect (θ) by the stratifier, dummy variables indicating membership are included. The degrees of freedom of the test statistic are the number of *α *parameters that are free under the no restrictions case *times *(*n *- 1) minus the number of dummy variable indicating the membership, where **n **is the number of groups over which we are testing the differences. An alternative approach is to consider the test statistic -2(*L*^*R - *^*L*^*Rτ *^), where we compare the (log) likelihood ratio of the fully restricted likelihood function and the partially restricted likelihood function (with cut-points held constant).

### Data

The data are from the Indian National Sample Survey of 1995-96. The survey collected information on SRH for all elderly (aged 60 years and above) in the households surveyed. It also collected information on what we designate objective measures of health of the elderly members of the household - such as information on events resulting in hospitalization, chronic illness, and disability. We consider hospitalization an objective measure of health because it is a discrete event unlikely to be easily forgotten or influenced by cultural and other factors driving the reporting of less serious types of illness, and reflects a significant shock to the health of the individual. Of course, income, timing, and the distance to health care facilities might lead to a seriously ill person foregoing hospitalization, and limiting the focus solely to hospital stays in the last one year as per the survey instrument would rule out individuals hospitalized in previous years.

One way to address the concern above using information on hospitalization would be to include information on chronic ailments such as heart disease, hypertension, cancers, diabetes, arthritis, and so forth that are of a longer-term nature. The National Sample Survey included this information in two forms, starting with conditions that were manifested in acute illness resulting in either hospitalization in the last one year or illness in the two weeks preceding the survey. A separate section for the elderly specifically inquired whether elderly respondents currently experienced these conditions, whether or not with a recent acute manifestation. In the paper, we report results using the second set of indicators of chronic conditions. Our results are not dependent, however, on whether we use indicators of chronic conditions with a recent acute manifestation or without.

We also included information on four indicators of disability among household members related to movement, sight, hearing, and speech. In general, indicators of disability, especially in self-assessments based on how difficult respondents find it to move, see, hear, and so forth, can be problematic and subject to the same difficulties in comparative assessments [10,11]. However, in the case of the National Sample Survey, indicators of disability can be treated as objective indicators of specific dimensions of health of the elderly population. There are two reasons for doing so. First, the survey inquiries relate to features of disability that go beyond a purely subjective assessment. For instance, the NSS defines disability in terms of impairments. Locomotive disability is defined as physical deformity even if it does not influence mobility. The definition also includes loss of activity of part of the hand or leg due to amputation, paralysis, or deformity. This can be directly observed by the interviewer. Visual disability in the survey is defined as when a person has no light perception and cannot correctly count the fingers of the hand from a distance of 10 feet in broad daylight (with/without spectacles). Second, a simple (1 if disabled, 0 if not) cut-off rule that respondents used ought to further eliminate any reporting biases at the upper and lower ends of the disability range.

Even when impairments are self-reported, as is likely in the case of visual acuity in the NSS, it is worth reflecting on the findings of the labor economics literature that commonly uses self-reported disability indicators in empirical estimates of labor supply models. Typically in this literature, the addition of other objective (clinically defined) health measures adds very little to the explanatory power of analyses [18]. Some researchers have criticized the validity of self-reported disability measures and argued that people may overreport disability to justify their difficulties in the labor market [19-22]. Other studies, however, have found little evidence of endogeneity of self-reported disability measures and labor force participation [23,24]. In our study, moreover, the validity of disability measures is not compromised by this justification hypothesis as the National Sample Survey for India did not explore questions of health and retirement in the same survey. Given the extremely limited nature of social protection programs, there is no real incentive for individuals to overreport disability.

The use of self-reported measures of specific disabilities to circumvent issues of rationalization in global measures of health is well-supported in the literature. For instance, even though Bound and colleagues argued against the use of global questions such as "How would you rate your health?" they assessed measures of limitation in physical function to be less susceptible to measurement and endogeneity problems [21]. Researchers have found that self-reported disability, when assessed by independent verification, provides a good description of true status [18,25,26]. Guralnik et al. also showed that self-report of disability was strongly associated with performance in a physical test among the elderly [27]. Moreover, Bailis et al., using data from various rounds of the longitudinal population health survey in Canada in the 1990s, found that self-reported aggregate health measures responded not only to changes in individuals' physical and mental health, but also to their intentions/expectations about their health behaviours in the future [28], a finding supported by [29] that used longitudinal data on health among adolescents for the United States. For these reasons, we expect disability measures in the NSS to provide a closer proxy to the true health of the elderly than the SRH. Moreover, generic instruments commonly used in measuring individual health status, such as Health Utility Index and EQ-5D, are all based on reporting of specific domains of functional limitations, such as mobility, hearing, vision, etc. These instruments are employed in various economic studies, clinical trials, and cost-effectiveness analyses to indicate objective health status and their validity, reliability, and comparability are widely accepted.

In addition to the objective indicators of health *H*^*o *^used in this paper, we also assumed the region of residence, sex, educational attainment, and rural-urban residence to influence the mapping from *H*^*o *^to self-assessed health *H*^*S *^and the cut-points used by individuals. We categorized states into four regions - North, East, South, and West based on geographic location (Table 1). Sex and rural-urban residence are defined as binary variables. Current location was used rather than individuals' original place of birth, both because the NSS data do not collect such information and also because of strong neighborhood effects on self-reported health documented in the literature [30,31]. Our indicator of educational attainment dichotomizes respondents into those with at least primary education and those with less than primary education. We chose the threshold of having completed primary education because several studies have shown that primary schooling offers the highest economic returns at the margin [32] and presumably the largest impact on attitudes and behavior. Unfortunately, experimenting with higher thresholds for education proved difficult because doing so drastically reduced the number of observations in some of the subgroups. Where the number of observations was sufficient to achieve statistical power, our findings remained unaffected.

**Table 1:** List of states by region

Region	States
North	Chandigarh, Delhi, Jammu & Kashmir, Haryana, Himachal Pradesh, Madhya Pradesh, Punjab, Rajasthan, Uttaranchal, Uttar Pradesh

East	Arunachal Pradesh, Assam, Bihar, Chhattisgarh, Jharkhand, Manipur, Meghalaya, Mizoram, Nagaland, Orissa, Sikkim, Tripura, West Bengal

South	Andaman & Nicobar, Andhra Pradesh, Karnataka, Kerala, Lakshadweep, Pondicherry, Tamil Nadu

West	Dadra & Nagar Haveli, Daman & Diu, Goa, Gujarat, Maharashtra

## Results

Descriptive statistics related to the elderly (60 years and above) are presented in Table 2. The sample size of the elderly in the 52^nd ^round of NSS was 33,940. The mean age of the sample elderly was 68 years, and the number of females in the sample is slightly more than the number of males. More than 70 percent of the elderly resided in rural areas. About one-fifth of the elderly in the sample had completed their primary education, with the elderly in the South and West regions having higher educational attainment than in other regions. Their average annual per capita consumption expenditure was INR 4,700 (about US$144), ranging from INR 4,000 rupees in the Eastern region to INR 5,400 in the West. Ties within the family and filial support are generally strong: about 78% of the elderly lived with their children, and only about 14% of the elderly lived alone (or with their spouse). Even among those living alone or with their spouse, a majority had children, grandchildren, or siblings staying in the same village or town, suggesting that (at least in 1995-96) family support systems for the Indian elderly were uniformly strong.

**Table 2:** Summary of variables and socioeconomic characteristics

Survey	Total	North	East	South	West
Sample size	33,940	12,545	8,692	7,795	4,908
Mean age	67.80	68.11	67.62	67.54	67.71
Male (%)	48.69	48.56	51.93	47.18	46.89
Urban (%)	21.92	18.45	17.93	25.58	30.09
***Socioeconomic characteristics***					
Primary education completed (%)	18.89	14.45	19.26	23.10	22.53
Avg. monthly per capita consumption expenditure (rupee)	389.87	393.20	336.71	391.96	451.79
Scheduled caste or scheduled tribe (%)	23.75	26.06	26.80	17.13	24.30
***Health variables***					
SRH- Excellent (%)	1.58	1.77	1.43	1.29	1.80
SRH- Good (%)	7.44	7.08	8.06	6.52	8.88
SRH- Fair (%)	71.65	72.03	67.23	73.38	74.11
SRH- Poor (%)	19.33	19.12	23.28	18.82	15.21
With one or more common chronic diseases^1 ^(%)	44.93	41.03	47.41	49.44	43.34
With one or more disabilities^2 ^(%)	36.07	35.76	36.85	34.59	37.80
Being hospitalized in the last year (%)	4.00	2.55	2.75	6.60	5.17

^1^The list of common chronic diseases includes joint disease, hypertension, heart disease, diabetes and cancer

^2^Including disabilities in visual, speech, hearing and motor function

The main variable of interest in our study is responses to the question on elderly perceptions of their own current health status. The response categories were excellent (*SRH = 4)*, good (*SRH = 3*), fair (*SRH = 2*), and poor (*SRH = 1*). In terms of the notation used in the statistical model of the previous section, this measure corresponds to *H*^*S*^. We see that the large majority of the elderly reported being in fair health (71.7 percent) and another 19.3 percent in poor health. Our sample data also confirm the findings of Amartya Sen [17], who observed that the population residing in the Southern region (including Kerala) had a lower proportion of individuals self-reporting to be in excellent or good health (7.8%) than the Eastern region (9.5%), which includes Bihar and is characterized by much lower levels of economic status and educational attainment. The prevalence of hospital stays, chronic conditions, and disabilities are also summarized in Table 2.

The empirical strategy of the previous section was applied to data on the elderly from the 52nd round of the National Sample Survey. We chose four factors to stratify our sample into homogeneous groups: gender, education, rural-urban residence, and region. In a developing country like India, where the life experience and social expectations of the two sexes are different, it is possible that males and females do not have the same expectations when assessing their health. The rationale behind education as a stratification device is also rather straightforward. Education likely shapes people's attitudes and perceptions toward their surroundings and themselves. Even though it has not been shown to affect cut-point differences in previous analyses [11], the substantial impact of primary education on health knowledge in developing countries documented in the literature warrants its inclusion. Hence, we divided the elderly into two groups, based on whether or not they completed their primary education. Economic status (in the form of consumption expenditure per capita) is not used for stratification because income and education were highly correlated, and using both income and education would have resulted in overstratification and limited sample sizes. Lastly, we used rural-urban residence and region as factors that would affect expectations of health, mainly because they are likely to be closely correlated with the living environment and other unobservable factors in the local context. In India, this is especially important owing to the considerable cultural differences (including language) that exist across regions.

Our results are presented in Tables 3, 4, 5 and 6. First, we tested whether the region of residence affected people's reporting behavior. Again, we should emphasize that what is likely at work is not the region per se but other commonly unobservable factors that are closely associated with where individuals live, such as ethnicity, language, and cultural practices. We separated the sample into homogeneous subgroups by gender, education, rural-urban residence, and region. As shown in Table 3, region generally has a significant impact on how women perceive their health (as indicated by SRH).

**Table 3:** Test for differential response by region of residence

**H**^**0**^	Males				Females			
	**Rural**		**Urban**		**Rural**		**Urban**	
	**Less than primary education**	**Primary education completed**	**Less than primary education**	**Primary education completed**	**Less than primary education**	**Primary education completed**	**Less than primary education**	**Primary education completed**

Log likelihood: North	-2459.77	-391.29	-561.68438	-762.63	-2810.45	-79.89	-1183.61	-307.07
Log likelihood: East	-1874.81	-471.61	-377.78396	-600.70	-2102.21	-56.35	-801.69	-291.29
Log likelihood: South	-947.86396	-387.01	-394.37561	-661.10	-1320.54	-169.58	-876.94	-329.00
Log likelihood: West	-598.91288	-125.49	-228.73973	-586.81	-801.73	-18.70	-551.19	-330.72
Log likelihood sum (unrestricted model, *L*^U^)	-5881.35	-1375.40	-1562.58	-2611.23	-7034.94	-324.52	-3413.43	-1258.08
Log likelihood restricted model (*L*^R^)	-5910.92	-1390.14	-1577.21	-2639.0483	-7083.58	-347.71777	-3446.14	-1284.52
*X*^*2*^*-first stage *test statistic: -2*(*L*^R^-*L*^U^)	59.14	29.49	29.25	55.64	97.28	46.39	65.41	52.88
Sample size	7694	1783	2144	3428	9465	476	4710	1759
***P-*value (24 degrees of freedom)**^**1**^	**8.4 × 10**^**-5**^	**0.202**	**0.211**	**0.0003**	**8.5.8 × 10**^**-11**^	**0.004**	**1.1 × 10**^**-5**^	**0.0006**
								
Log likelihood partially-restricted model (*L*^Rα^)	-5898.58	n.a.	n.a.	-26.18.29	-7067.62	-330.00	-3431.05	-1269.87
*X*^*2*^-test statistic for cut-point differences: -2*( *L*^Rα^-*L*^U^)	34.46	n.a.	n.a.	14.13	65.36	10.95	35.24	23.59
***P-*value (6 degrees of freedom)**^**2**^	**5.5 × 10**^**-6**^	**n.a**.	**n.a**.	**0.028**	**3.7 × 10-12**	**0.090**	**3.9 × 10**^**-6**^	**0.0006**
*X*^*2*^-test statistics for g (.): -2*( *L*^R^-*L*^Rα^)	24.68	n.a.	n.a.	41.51	31.92	35.44	30.17	29.29
***P-*value (18 degrees of freedom)**^**3**^	**0.134**	**n.a**.	**n.a**.	**0.001**	**0.022**	**0.008**	**0.036**	**0.045**

^1^6 parameters for H^o ^(α) and 3 cut-points (τ) were estimated the unrestricted model for four subgroups (9 × 4 = 36); 6 parameters for H^o ^(α), 3 parameters for membership (θ) and 3 cut-points (τ) were estimated for the restricted model. The degree of freedom is equal to the difference in the number of estimated parameters between these two models (36-12 = 24).

^2 ^30 parameters, including 6 parameters for H^o ^(α) for the four subgroups (6 × 4 = 24), 3 parameters for membership (θ) and 3 cut-points (τ) were estimated for the partially-restricted model. The degree of freedom is equal to the difference of number of estimated parameters between unrestricted and partially-restricted models (36-30 = 6).

^3 ^The degree of freedom is equal to the difference of number of estimated parameters between partially-restricted models and restricted models (30-12 = 18).

**Table 4:** Test for differential response by gender

	Less than primary education				Primary education completed			
	North	East	South	West	North	East	South	West
*Rural*								
Log likelihood: Female	-2810.45	-2102.21	-1320.54	-801.73	-79.89	-56.35	-169.58	-18.70
Log likelihood: Male	-2459.77	-1874.81	-947.86	-598.91	-391.29	-471.61	-387.01	-125.49
Log likelihood sum (unrestricted model, *L*^U^)	-5270.22	-3977.02	-2268.41	-1400.64	-471.18	-527.96	-556.59	-144.19
Log likelihood restricted model (*L*^R^)	-5275.57	-3982.81	-2271.41	-1404.48	-474.22	-543.43	-558.48	-148.23
*X*^*2*^*-first stage *test statistic: -2*( *L*^R^-*L*^U^)	10.70	11.59	6.01	7.68	6.08	30.95	3.78	8.09
Sample size	7185	4827	3113	2034	619	685	736	219
***P-*value (8 degrees of freedom)**^**1**^	**0.22**	**0.17**	**0.65**	**0.47**	**0.64**	**0.0001**	**0.88**	**0.43**
Log likelihood partially-restricted model (*L*^Rα^)	n.a.	n.a.	n.a.	n.a.	n.a.	-535.07	n.a.	n.a.
*X*^*2*^-test statistic for cut-point shift: -2*( *L*^Rα^-*L*^U^)	n.a.	n.a.	n.a.	n.a.	n.a.	14.22	n.a.	n.a.
***P-*value (2 degrees of freedom)**^**2**^	**n.a**.	**n.a**.	**n.a**.	**n.a**.	**n.a**.	**0.001**	**n.a**.	**n.a**.
*X*^*2*^-test statistic for g (.) function: -2*( *L*^R^-*L*^Rα^)	n.a.	n.a.	n.a.	n.a.	n.a.	16.74	n.a.	n.a.
***P-*value (6 degrees of freedom)**^**3**^	**n.a**.	**n.a**.	**n.a**.	**n.a**.	**n.a**.	**0.010**	**n.a**.	**n.a**.
								
*Urban*								
Log likelihood: Female	-1183.61	-801.69	-876.94	-551.19	-307.07	-291.29	-329.00	-330.72
Log likelihood: Male	-561.68	-377.78	-394.38	-228.74	-762.63	-600.70	-661.10	-586.81
Log likelihood sum (unrestricted model, *L*^U^)	-1745.30	-1179.47	-1271.32	-779.93	-1069.69	-891.99	-990.10	-917.53
Log likelihood restricted model (*L*^R^)	-1746.76	-1183.38	-1283.50	-784.23	-1071.20	-895.77	-993.93	-919.59
*X*^*2*^*-first stage *test statistic: -2*( *L*^R^-*L*^U^)	2.92	7.83	24.37	8.58	3.01	7.57	7.67	4.13
Sample size	2406	1439	1866	1143	1471	1066	1474	1176
***P-*value (8 degrees of freedom)**^**1**^	**0.94**	**0.45**	**0.002**	**0.38**	**0.93**	**0.48**	**0.47**	**0.85**
Log likelihood partially-restricted model (*L*^Rα^)	n.a.	n.a.	-1272.32	n.a.	n.a.	n.a.	n.a.	n.a.
*X*^*2*^-test statistic for cut-point shift: -2*( *L*^Rα^-*L*^U^)	n.a.	n.a.	2.00	n.a.	n.a.	n.a.	n.a.	n.a.
***P-*value (2 degrees of freedom)**^**2**^	**n.a**.	**n.a**.	**0.368**	**n.a**.	**n.a**.	**n.a**.	**n.a**.	**n.a**.
*X*^*2*^-test statistic for g (.) function: -2*( *L*^R^-*L*^Rα^)	n.a.	n.a.	22.37	n.a.	n.a.	n.a.	n.a.	n.a.
***P-*value (6 degrees of freedom)**^**3**^	**n.a**.	**n.a**.	**0.0010**	**n.a**.	**n.a**.	**n.a**.	**n.a**.	**n.a**.

^1^6 parameters for H^o ^(α) and 3 cut-points (τ) were estimated the unrestricted model for two subgroups (9 × 2 = 18); 6 parameters for H^o ^(α), 1 parameters for membership (θ) and 3 cut-points (τ) were estimated for the restricted model. The degree of freedom is equal to the difference in the number of estimated parameters between these two models (18-10 = 8).

^2 ^16 parameters, including 6 parameters for H^o ^(α) for the two subgroups (6 × 2 = 12), 1 parameters for membership (θ) and 3 cut-points (τ) were estimated for the partially-restricted model. The degree of freedom is equal to the difference of number of estimated parameters between unrestricted and partially-restricted models (18-16 = 2).

^3 ^The degree of freedom is equal to the difference of number of estimated parameters between partially-restricted models and restricted models (16-10 = 6).

**Table 5:** Test for differential response by education

	Rural				Urban			
	North	East	South	West	North	East	South	West
*Males*								
Log likelihood: <primary	-2459.77	-1874.81	-947.86	-598.54	-561.68	-377.78	-394.38	-228.74
Log likelihood: primary+	-391.29	-471.61	-387.01	-125.45	-762.63	-600.70	-661.10	-586.81
								
Log likelihood sum (unrestricted model, *L*^U^)	-2851.06	-2346.41	-1334.88	-723.99	-1324.31	-978.48	-1055.47	-815.55
Log likelihood restricted model (*L*^R^)	-2856.40	-2350.25	-1338.57	-729.46	-1325.46	-986.37	-1060.74	-817.75
*X*^*2*^*-first stage *test statistic: -2*( *L*^R^-*L*^U^)	10.67	7.68	7.39	10.94	2.29	15.77	10.53	4.41
Sample size	3793	2820	1832	1032	1820	1199	1499	1054
***P-*value (8 degrees of freedom)**^**1**^	**0.22**	**0.47**	**0.50**	**0.21**	**0.97**	**0.05**	**0.23**	**0.82**
Log likelihood partially-restricted model (*L*^Rα^)	n.a.	n.a.	n.a.	n.a.	n.a.	n.a.	n.a.	n.a.
*X*^*2*^-test statistic for cut-point shift: -2*( *L*^Rα^-*L*^U^)	n.a.	n.a.	n.a.	n.a.	n.a.	n.a.	n.a.	n.a.
***P-*value (2 degrees of freedom)**^**2**^	**n.a**.	**n.a**.	**n.a**.	**n.a**.	**n.a**.	**n.a**.	**n.a**.	**n.a**.
*X*^*2*^-test statistic for g (.) function: -2*( *L*^R^-*L*^Rα^)	n.a.	n.a.	n.a.	n.a.	n.a.	n.a.	n.a.	n.a.
***P-*value (6 degrees of freedom)**^**3**^	**n.a**.	**n.a**.	**n.a**.	**n.a**.	**n.a**.	**n.a**.	**n.a**.	**n.a**.
								
*Females*								
Log likelihood: <primary	-2810.45	-2102.21	-1320.54	-801.73	-1183.61	-801.69	-876.94	-551.19
Log likelihood: primary+	-79.89	-56.35	-169.58	-18.70	-307.07	-291.29	-329.00	-330.72
								
Log likelihood sum (unrestricted model, *L*^U^)	-2890.34	-2158.56	-1490.12	-820.43	-1490.68	-1092.98	-1205.94	-881.91
Log likelihood restricted model (*L*^R^)	-2893.46	-2175.41	-1495.02	-825.57	-1494.84	-1098.79	-1213.65	-884.54
*X*^*2*^*-first stage *test statistic: -2*( *L*^R^-*L*^U^)	6.23	33.69	9.81	10.27	8.32	11.63	15.42	5.24
Sample size	4011	2692	2017	1221	2057	1306	1841	1265
***P-*value (8 degrees of freedom)**^**1**^	**0.62**	**4.6 × 10-5**	**0.28**	**0.25**	**0.40**	**0.17**	**0.05**	**0.73**
Log likelihood partially-restricted model (*L*^Rα^)	n.a.	-2167.19	n.a.	n.a.	n.a.	n.a.	n.a.	n.a.
*X*^*2*^-test statistic for cut-point shift: -2*( *L*^Rα^-*L*^U^)	n.a.	17.26	n.a.	n.a.	n.a.	n.a.	n.a.	n.a.
***P-*value (2 degrees of freedom)**^**2**^	**n.a**.	**0.0002**	**n.a**.	**n.a**.	**n.a**.	**n.a**.	**n.a**.	**n.a**.
*X*^*2*^-test statistic for g (.) function: -2*( *L*^R^-*L*^Rα^)	n.a.	16.43	n.a.	n.a.	n.a.	n.a.	n.a.	n.a.
***P-*value (6 degrees of freedom)**^**3**^	**n.a**.	**0.012**	**n.a**.	**n.a**.	**n.a**.	**n.a**.	**n.a**.	**n.a**.

^1^6 parameters for H^o ^(α) and 3 cut-points (τ) were estimated the unrestricted model for two subgroups (9 × 2 = 18); 6 parameters for H^o ^(α), 1 parameters for membership (θ) and 3 cut-points (τ) were estimated for the restricted model. The degree of freedom is equal to the difference in the number of estimated parameters between these two models (18-10 = 8).

^2 ^16 parameters, including 6 parameters for H^o ^(α) for the two subgroups (6 × 2 = 12), 1 parameters for membership (θ) and 3 cut-points (τ) were estimated for the partially-restricted model. The degree of freedom is equal to the difference of number of estimated parameters between unrestricted and partially-restricted models (18-16 = 2).

^3 ^The degree of freedom is equal to the difference of number of estimated parameters between partially-restricted models and restricted models (16-10 = 6).

**Table 6:** Test for differential response by sector

	Less than primary education				Primary education completed			
	North	East	South	West	North	East	South	West
*Males*								
Log likelihood: rural	-2459.77	-1874.81	-947.86	-598.91	-391.29	-471.61	-387.01	-125.49
Log likelihood: urban	-561.68	-377.78	-394.38	-228.74	-762.63	-600.70	-661.10	-586.81
Log likelihood sum (unrestricted model, *L*^U^)	-3021.45	-2252.59	-1342.24	-827.65	-1153.92	-1072.30	-1048.11	-712.29
Log likelihood restricted model (*L*^R^)	-3025.87	-2256.75	-1347.63	-833.03	-1155.66	-1081.02	-1050.42	-716.86
*X*^*2*^*-first stage *test statistic: -2*( *L*^R^-*L*^U^)	8.83	8.32	10.78	10.74	3.49	17.43	4.63	9.14
Sample size	4069	2713	1888	1168	1544	1306	1443	918
***P-*value (8 degrees of freedom)**^**1**^	**0.36**	**0.40**	**0.21**	**0.22**	**0.90**	**0.03**	**0.80**	**0.33**
Log likelihood partially-restricted model (*L*^Rα^)	n.a.	n.a.	n.a.	n.a.	n.a.	-1072.72	n.a.	n.a.
*X*^*2*^-test statistic for cut-point shift: -2*( *L*^Rα^-*L*^U^)	n.a.	n.a.	n.a.	n.a.	n.a.	0.84	n.a.	n.a.
***P-*value (2 degrees of freedom)**^**2**^	**n.a**.	**n.a**.	**n.a**.	**n.a**.	**n.a**.	**0.659**	**n.a**.	**n.a**.
*X*^*2*^-test statistic for g (.) function: -2*( *L*^R^-*L*^Rα^)	n.a.	n.a.	n.a.	n.a.	n.a.	16.59	n.a.	n.a.
***P-*value (6 degrees of freedom)**^**3**^	**n.a**.	**n.a**.	**n.a**.	**n.a**.	**n.a**.	**0.011**	**n.a**.	**n.a**.
								
*Females*								
Log likelihood: rural	-2810.45	-2102.21	-1320.54	-801.73	-79.89	-56.35	-169.58	-18.70
Log likelihood: urban	-1183.61	-801.69	-876.94	-551.19	-307.07	-291.29	-329.00	-330.72
Log likelihood sum (unrestricted model, *L*^U^)	-3994.06	-2903.90	-2197.49	-1352.93	-386.96	-347.64	-498.58	-349.42
Log likelihood restricted model (*L*^R^)	-3998.27	-2911.38	-2210.64	-1360.39	-391.51	-365.76	-503.08	-354.41
*X*^*2*^*-first stage *test statistic: -2*( *L*^R^-*L*^U^)	8.42	14.97	26.32	14.93	9.09	36.24	9.00	9.98
Sample size	5522	3553	3091	2009	546	445	767	477
***P-*value (8 degrees of freedom)**^**1**^	**0.39**	**0.06**	**9.27 × 10-04**	**0.06**	**0.33**	**1.59 × 10-05**	**0.34**	**0.27**
Log likelihood partially-restricted model (*L*^Rα^)	n.a.	n.a.	-2205.52	n.a.	n.a.	-355.21	n.a.	n.a.
*X*^*2*^-test statistic for cut-point shift: -2*( *L*^Rα^-*L*^U^)	n.a.	n.a.	16.08	n.a.	n.a.	15.14	n.a.	n.a.
***P-*value (2 degrees of freedom)**^**2**^	**n.a**.	**n.a**.	**0.0003**	**n.a**.	**n.a**.	**0.0005**	**n.a**.	**n.a**.
*X*^*2*^-test statistic for g (.) function: -2*( *L*^R^-*L*^Rα^)	n.a.	n.a.	10.24	n.a.	n.a.	21.10	n.a.	n.a.
***P-*value (6 degrees of freedom)**^**3**^	**n.a**.	**n.a**.	**0.115**	**n.a**.	**n.a**.	**0.002**	**n.a**.	**n.a**.

^1^6 parameters for H^o ^(α) and 3 cut-points (τ) were estimated the unrestricted model for two subgroups (9 × 2 = 18); 6 parameters for H^o ^(α), 1 parameters for membership (θ) and 3 cut-points (τ) were estimated for the restricted model. The degree of freedom is equal to the difference in the number of estimated parameters between these two models (18-10 = 8).

^2 ^16 parameters, including 6 parameters for H^o ^(α) for the two subgroups (6 × 2 = 12), 1 parameters for membership (θ) and 3 cut-points (τ) were estimated for the partially-restricted model. The degree of freedom is equal to the difference of number of estimated parameters between unrestricted and partially-restricted models (18-16 = 2).

^3 ^The degree of freedom is equal to the difference of number of estimated parameters between partially-restricted models and restricted models (16-10 = 6).

For males, the region of residence made a difference in health reporting behavior among two groups of men: rural men who had not completed their primary education and urban men who had completed their primary education. Robustness checks conducted by using only disability indicators as objective health indicators (results not shown) confirm these basic findings. Again, the underlying factor is the cut-point differences.

**Figure 1:**
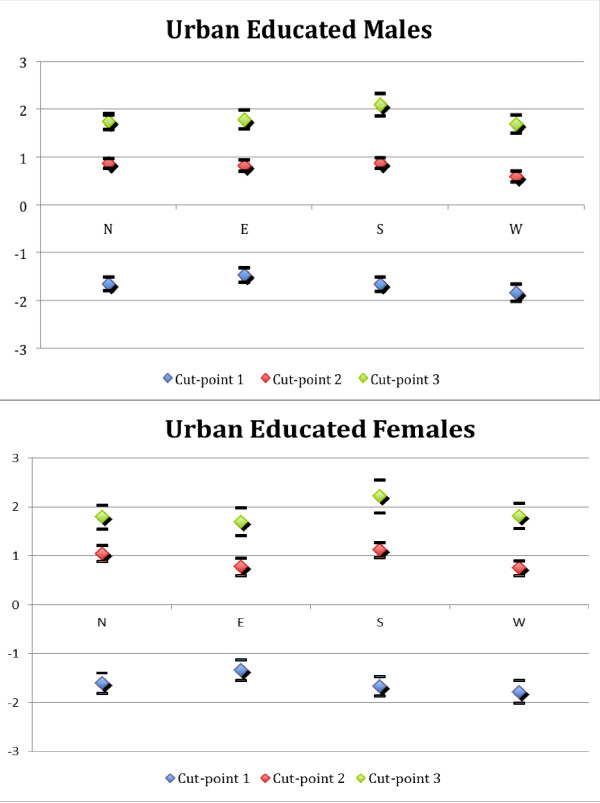
**SRH cut-points among urban population with at least primary education**. Note: Cut-point 1 is the cut-point between "poor" and "fair" health; cut-point 2 is the cut-point between "fair" and "good" health; and cut-point 3 is the cut-point between "good" and "excellent" health. Upper and lower bars indicate the upper and lower limits of the 95% confidence intervals of the estimates, respectively.

To visualize the cut-point differences and their effect on reporting heterogeneity among residents of different regions, we illustrate the cut-points of urban male and female elderly with at least primary education as an example in Figure 1.

Note in Figure 1 that cut-points can differ across groups in two ways: in the level and in the distance between cut-points within each group. Take the South and East regions as an example. Men and women in the Southern region had higher cut-points 2 and 3 than those in the Eastern region, meaning that at a given health status of fair or better, the elderly in the East would report a better health status than those living in the South. At the same time, the distances between cut-points are also wider in Southern region - it has a lower cut-point 1 compared to the Eastern region; therefore, compared with the elderly in the Eastern region, the former would tend not to report extreme values when assessing their own health.

Reporting heterogeneity in SRH can arise not only from cut-point shifts, but also due to differences in parameters of the *g*(.) function; that is, due to differences in the influence of objective health measures (the coefficients α in the above model specification). This appears to be the case for women in our sample.

We also tested for differences in cut-points and *α *across gender and by educational achievement (see Tables 4 and 5). Given our previous finding that cut-points and the parameters of the H* equation vary by region, we did not aggregate the observations across different regional groups. In fact, in the remainder of this analysis, the sample remained disaggregated as 16 subgroups. Lindeboom and van Doorslaer tested for reporting heterogeneity across language groups and found that language made a difference in only a few subgroups. Hence, they aggregated the sample across language groups in the later tests for the impact of other factors. In order to ensure that we did not falsely reject the null hypothesis because of the aggregation of subgroups, we chose the conservative strategy and kept the sample in homogeneous disaggregated groups. As seen in Tables 4 and 5, we found little evidence of differences in cut-points and *α *by gender or educational status.

In Table 6 we report the results of tests for differences by rural and urban residence. In some cases, namely elderly that had completed primary education in the Eastern region and elderly women with less than primary education in the Southern region, there are rural-urban differences. In general, though, there is no evidence of reporting heterogeneity between rural and urban areas.

Table 7 presents estimates of the vector α of parameters that characterize the relationship between objective indicators of health and latent true health H* for a specific case, rural females with less than primary education: estimates from ordered probit models for different regions, with and without the restriction of equal cut-point and true health coefficients. These estimates are consistent with what one might expect - the coefficients are of the right sign (indicators of chronic conditions, hospitalization, and disability are negatively related to the indicator of true health H*) and statistically significant except for speech disability. The fixed-effect estimates of the regional dummy variables in the restricted specification illustrate the regional impact on the distribution of elderly SRH via some combination of a direct impact on true health and a parallel shift in cut-points. On average, the regional impact was highest among elderly SRH in the West region, followed by the South and the North. Note from the results on the unrestricted models that the effect of disability measures on true health varied across disability types, with motor function having a larger effect than, say, speech, and that there was variation in these coefficients across the different regions.

**Table 7:** Estimates of health effects in restricted and unrestricted models^1^

	Restricted Model	Unrestricted Model			
		
		*North*	*East*	*South*	*West*
**Chronic dz.**	**-0.35**	**-0.30**	**-0.34**	**-0.35**	**-0.51**
	(0.031)**	(0.055)**	(0.048)**	(0.061)**	(0.101)**
**Hospitalization**	**-0.33**	**-0.38**	**-0.19**	**-0.36**	**-0.30**
	(0.052)**	(0.087)**	(0.111)	(0.101)**	(0.124)*
**Visual**	**-0.40**	**-0.28**	**-0.53**	**-0.48**	**-0.38**
	(0.030)**	(0.045)**	(0.054)**	(0.063)**	(0.075)**
**Speech**	**0.08**	**0.13**	**-0.03**	**0.08**	**0.24**
	(0.067)	(0.108)	(0.117)	(0.180)	(0.179)
**Hearing**	**-0.30**	**-0.41**	**-0.26**	**-0.23**	**-0.21**
	(0.037)**	(0.059)**	(0.062)**	(0.087)**	(0.102)*
**Motor**	**-0.73**	**-0.70**	**-0.79**	**-0.76**	**-0.73**
	(0.042)**	(0.064)**	(0.082)**	(0.094)**	(0.116)**
**North**	**0.05**	-	-	-	-
	(0.031)				
**South**	**0.06**	-	-	-	-
	(0.037)				
**West**	**0.12**	-	-	-	-
	(0.043)**				

* = Significance at 0.05 level ** = Significance at 0.01 level

^1^The estimates are based on analysis of the rural females with less than primary education

As a further robustness check, we sought to assess whether sample sizes in our subgroups for education and gender were large enough to allow for sufficient power in the detection of differences in cut-points and coefficients on our measures of objective health. Because no closed-form solutions for assessing the power of the test statistic were available, we performed Monte Carlo simulations to do so. Our results do not lend support to the idea that statistical insignificance across subgroups based on gender and education was the result of inadequate sample size. As an illustration, to detect a relatively small (10%) difference between the lowest cut-point for males and that of females at the 5 percent level of significance, we estimated the power of the test to be approximately 50% with a sample size of 200. The power increased to 76% with a sample size of 400 and more than 80% with a sample size of 600. This was satisfied in all of the stratified groups, except for one case - individuals who had completed primary education in the rural West.

As another robustness check of our findings, we also conducted a series of analyses using current economic status (whether above/below per capita consumption expenditure) and caste status (scheduled castes/tribes and other) as stratifiers for socioeconomic status. We also applied the same empirical testing procedure to a different dataset, the 60^th ^round of the National Sample Survey. Similar to the 52^nd ^round, the 60^th ^round of NSS, which was conducted in 2004, also collected information on socioeconomic status, health status, and service utilization, as well as a special section on the elderly. Unfortunately, several questions on elderly disability and chronic conditions are missing in the 2004 survey, and thus do not allow a direct comparison of the results from the two surveys. Qualitatively, though, the results are similar: In most of the homogeneous subgroups, reporting behavior differs by the region of residence, and excepting a few groups, there was no statistical evidence to suggest that gender and education divisions influence the relationship between H* and SRH.

## Discussion and Conclusions

Comparable health measures across different populations are essential in understanding the distribution of health outcomes. It is also instrumental in assessing the impact of health policy interventions. SRH is a commonly used indicator of health in existing household surveys and has been shown to be predictive of future mortality. However, the susceptibility of SRH to influence by individuals' expectations complicates its interpretation and undermines its usefulness.

Our paper supports the thesis that the region of residence is associated with different cut-points and reporting behavior on health survey questions, although gender, education, and rural-urban residence do not appear to systematically compromise the comparability of self-reported health measures. The cut-points can differ in two ways: they can be systematically higher or lower, and the distance between cut-points, which reflects the degree of aversion to reporting extreme values, can vary as well. These findings are robust with different specifications of socioeconomic status or when we restrict the objective health indicators to only functional disabilities. In this, our finding lends limited support to the work of Sen [17] on differences in reported health between Kerala (located in South India) and Bihar (located in East India). As we demonstrated, the cut-points among elderly of different regions were different, and hence the regional distribution of health based on self-assessed indicators may not adequately capture true health differentials. This is illustrated by our findings in Figures 1 showing that the elderly with fair or better health status in the Southern region had higher cut-points and tended to underrate their health status This might help explain why the difference in health between the East and South regions was not larger despite the much better development and health achievement in the South region documented in the literature. Whether intraregional comparisons of SRH can be made is unclear, however. Further testing of our model via the construction of within-region subgroups was limited by the size of our sample. There is little doubt that the populations in the regions we chose are culturally more similar to each other than their counterparts in other regions in terms of language, colonial history, and Islamic influence. But it is difficult to argue in favor of complete within-region homogeneity and comparability owing to differences that are sometimes evident even across neighboring districts within a region [33].

There are several limitations to our study. First, the findings may be specific to the case of Indian elderly, and one needs to be careful in generalizing the findings. There might well be interactions between region and other elements of socioeconomic status; that is, education could affect health expectations in one place but not another. Second, the validity of the method employed hinges on the choice of observable objective health indicators (H^0^). The variables we used as objective indicators of health are self-reported, even if NSS defines disability in terms of specific impairments. In this, our analysis is similar to instruments that have previously been used to assess objective health status in the literature, such as the Health Utility Index. Of course, biomarkers could serve as ideal objective H^0^, but unless they cover a wide range, they would reflect a very specific and narrowed domain of health. To the extent that any health impacts associated with biomarkers are unobserved by the individuals in question, we should not expect them to change an individual's own understanding of true health. Capturing data on a huge array of biomarkers is also extremely difficult to execute in a large-scale survey. Finally, one could come up with a long list of potential factors that result in reporting heterogeneity. However, to stratify across finer groups would have reduced subgroup sample sizes to impractically small. These shortcomings apart, our study suggests that local contexts matter in people's expectations relating to their health. Even within the same country, people can be quite dissimilar in their health perceptions, and thus attempts to use SRH to compare the population health of different regions within countries ought to be conducted with caution.

## Competing interests

The authors declare that they have no competing interests.

## Authors' contributions

Both authors contributed to design, data manipulation, data analysis, interpretation of findings, and drafting of the manuscript. Both authors read and approved the final manuscript.
